# A pilot study evaluating the utility of commercially available antibodies for flow cytometric analysis of *Panthera* species lymphocytes

**DOI:** 10.1186/s12917-018-1717-4

**Published:** 2018-12-19

**Authors:** Tashnica Taime Sylvester, Sven David Charles Parsons, Paul David van Helden, Michele Ann Miller, Andre Gareth Loxton

**Affiliations:** NRF/DST Centre of Excellence for Biomedical Tuberculosis Research; South African Medical Research Council Centre for Tuberculosis Research; Division of Molecular Biology and Human Genetics, Faculty of Medicine and Health Sciences, Stellenbosch University, Cape Town, South Africa

**Keywords:** African lion, Flow cytometery, Immunophenotype, *Panthera leo*

## Abstract

**Background:**

The immune response against tuberculosis in lions is still poorly defined and our understanding is hampered by the lack of lion specific reagents. The process for producing antibodies against a specific antigen is laborious and not available to many research laboratories. As the search for antibody cross-reactivity is an important strategy for immunological studies in veterinary medicine, we have investigated the use of commercially available antibodies to characterize T cell subsets in African lions (*Panthera leo)*.

**Results:**

Commercially available antibodies were screened and investigated the influence of two different sample processing methods, as well as the effect of time delay on cell surface marker expression on lion lymphocytes. Using commercially available antibodies, we were able to identify CD4^+^, CD5^+^, CD8^+^, CD14^+^, CD25^+^, CD44^+^ and CD45^+^ T lymphocytes in samples obtained by density gradient centrifugation as well as red cell lysis of lion whole blood. Two distinct lymphocyte populations, which differed in size and phenotype, were observed in the samples processed by density gradient centrifugation.

**Conclusion:**

Commercially available antibodies are able to differentiate between T lymphocyte subsets including immune effector cells in African lion whole blood, and possibly give insight into unique specie phenotypes.

**Electronic supplementary material:**

The online version of this article (10.1186/s12917-018-1717-4) contains supplementary material, which is available to authorized users.

## Background

The immune system is a complex and multi-faceted system which includes components such as memory and self-regulation. Flow cytometry is a commonly used tool in immunological research, making use of fluorophore-labelled antibodies to detect specific proteins expressed intracellularly and on cell membranes [1]. This technique is used to monitor immune responses to infection, track the phenotypic and functional characteristics of antigen-specific cells, and has more recently been used for the routine clinical diagnosis, prognosis and monitoring of disease [1].

Understanding the influence of pathogens, such as *Mycobacterium bovis* and feline immunodeficiency virus (FIV), opportunistic organisms and environmental heterogeneity on wild lion populations is crucial as the loss of these apex predators can have devastating effects on the ecosystems they inhabit [2]. Despite this, there is currently little known about how health and immunity are shaped by *M. bovis* and FIV co-infection when the environment and the host and pathogen genotypes vary. Knowledge of comparative immunology of wildlife can further facilitate our understanding of the interactions between host responses, ecology, evolution and health [3]. Generally, wildlife populations have a high degree of genetic variation which affects the robustness of the immune response, possibly making investigations in these populations more reflective of what happens in humans compared to laboratory models [4]. However, research in non-model animal species, in particular those living in the wild [5] or wildlife species who may be reservoirs for disease, a lack of species specific reagents limit our current ability to easily characterize immune phenotypes in these species [6, 7]. For this reason the de novo manufacturing of reagents or the evaluation of cross-reactive antibodies from related domestic species are needed to study species such as wild felids. Because no commercial antibodies have specifically been produced for use in flow cytometry in lions, the investigation and characterization of immune cell phenotypes in this species remains challenging. Despite these issues, through the use of cross-reactive antibodies, researchers have shown that FIV infection in lions is associated with lower levels of circulating CD4-positive T cells [8–10] as well as an increase in the CD8β^high^ subset [9, 10].

The aim of this study was to identify commercially available antibodies that can be used for flow cytometry in *Panthera* species. Furthermore, we sought to compare the effects of density gradient centrifugation and red cell lysis of whole blood on the binding of these antibodies, as well as investigate the effect of time delay on the staining patterns of these antibodies.

## Results

**Fig. 1:**
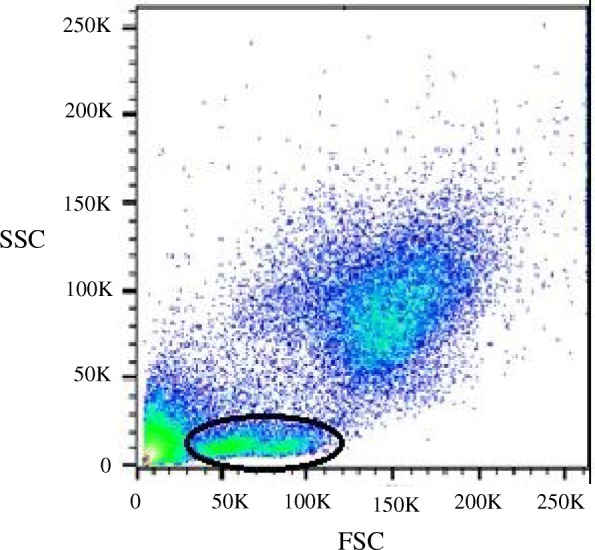
Representative flow cytometric dot plot of peripheral blood cell sorting. Forward scatter (FSC) versus side scatter (SSC) dot plots of PBMCs from a lion showing two distinct lymphocyte populations (total lymphocytes circled)

The scatter plots of SSC versus FSC for lion and tiger peripheral blood leukocytes revealed distinct cell populations similar to that of other mammals (Fig. 1). Based on these scatter parameters, the percentages of lymphocytes in PBMCs on day 1 were 33.0, 20.9% and 30.3 for lion 1, lion 2 and the tiger, respectively. The percentages of lymphocytes in RCLLs were 24.9, 17.8, and 10.6% in lion 1, lion 2 and the tiger, respectively. In scatterplots of PBMCs, but not RCLLs, from all three animals, small and large lymphocytes appeared as distinct populations (Fig. 1).

### Reactivity with lion leukocytes

All antibodies (described in Table 1) with the exception of CD4 (clone SK3), CD4 (clone RPA-T4), CD19 (clone SJ25C1), CD19 (clone MB19–1), CD62L (clone DREG-56) and IgM (clone G20–127) showed clear cross-reactivity with lion and tiger leukocyte populations in PBMCs (Additional file 1: Figure S1) and RCLLs (Additional file 1: Figure S2).

**Table 1:** Commercially available antibodies screened for cross-reactivity with African lion lymphocytes

Surface marker	Clone	Reactivity	Fluorophore	Company	Optimal conc (ug/ml)	
					PBMC	RCLL
CD5	f43	Feline/ Lion (PBMC)	PE	Southern Biotech, Birmingham, AL, USA	0.5	0.5
CD4	3-4f4	Feline	FITC	Southern Biotech	1.75	1.75
CD4	SK3	Human	APC	BD Biosciences	NB^a^	NB
CD4	RPA-T4	Human	V500	BD Biosciences	NB	NB
CD8β	fCD8	Feline	FITC	Southern Biotech	0.25	1.75
			PE	Southern Biotech	0.25	3.5
CD11B	M1/70	Mouse, Human	PERCP-CY5.5	Abcam, Cambridge, UK	1.75	3.5
CD14	61D3	Human, Cynomolgus, Canine, Hooded Seal	APC-CY7	Southern Biotech	3.5	1.75
CD19	SJ25C1	Human	BV510	BD Biosciences	NB	NB
CD19	MB19–1	Mouse, Canine	APC-CY7	Life Technologies, Carlsbad, CA, United States	NB	NB
CD25	P4A10	Canine	Biotin/HRP-HV500	Bio-rad, Hercules, CA, United States	0.5	1.75
CD44	IM7	Mouse	PERCP-CY5.5	Southern Biotech	5	5
CD45R	RA3-6B2	Mouse, Human	APC-CY7	Southern Biotech	2.5	2.5
CD62L	DREG-56	Human, Chimpanzee, Cattle	V450	BD Biosciences	NB	NB
IgM	G20–127	Human	BV510	BD Biosciences	NB	NB

^a^Antibodies that did not bind to lion *PBMC* Peripheral blood mononuclear cells or *RCLLs* Red cell lysis leukocytes are indicated in the optimal concentration column as *NB* Non-binding

### Staining pattern

**Fig. 2:**
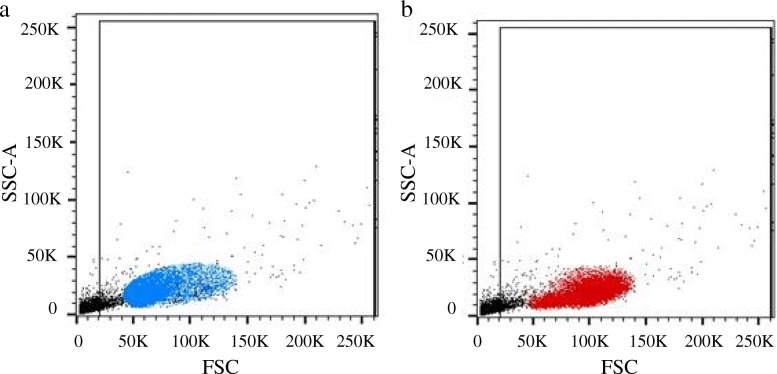
Representative flow cytometeric scatter plots of PBMCs from a lion indicating the occurrence of two distinct lymphocyte populations. The smaller lymphocytes are mainly comprised of **a** CD4+ cells, whereas **b** CD8+ cells make up the larger lymphocytes

**Fig. 3:**
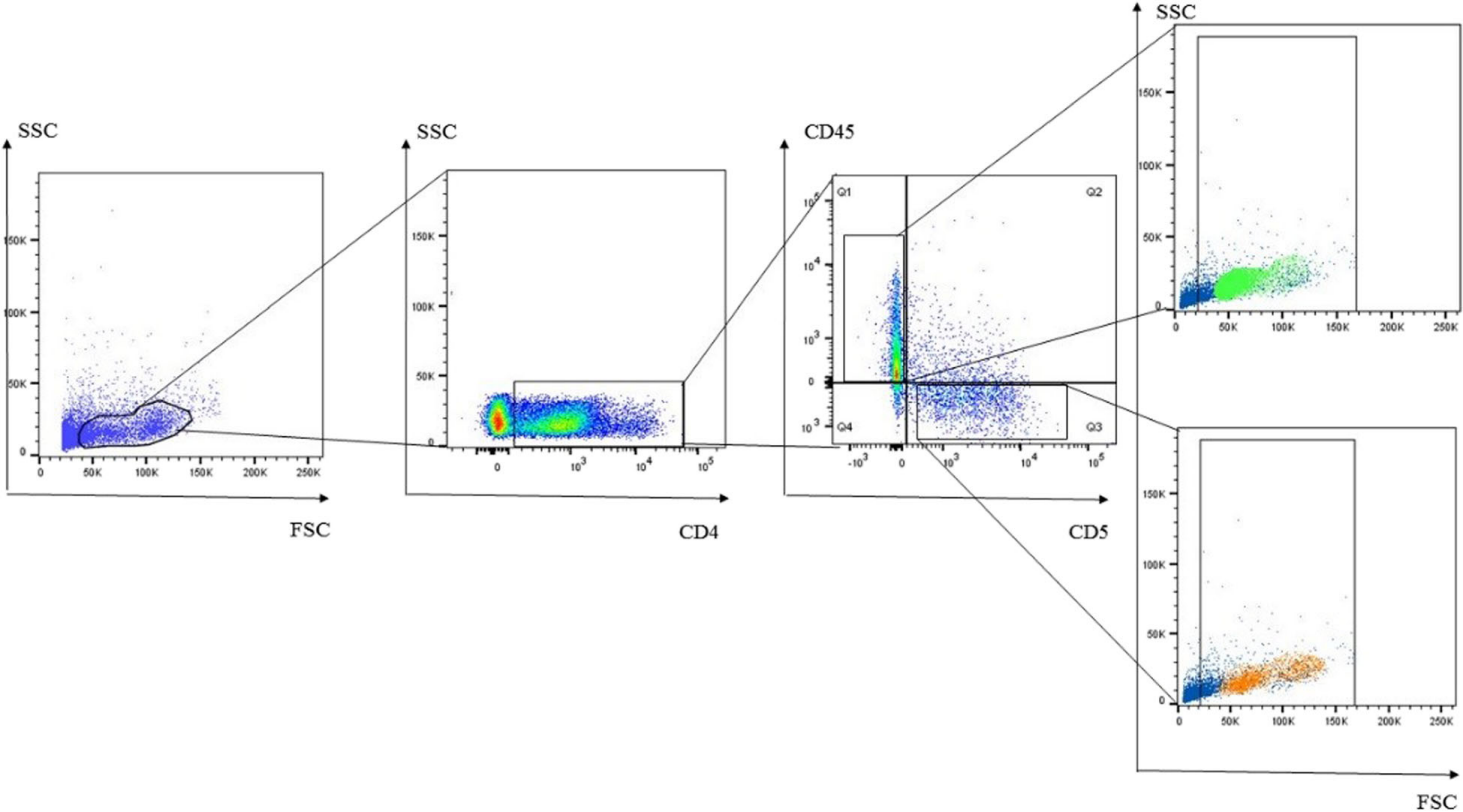
Representative flow cytometric dot plot of lion PBMCs. CD4 + CD5 + CD45- and CD4 + CD5-CD45+ lymphocytes were selected for back gating to establish their distribution across the two distinct lymphocyte populations observed on the FSC versus SSC dot plots

**Fig. 4:**
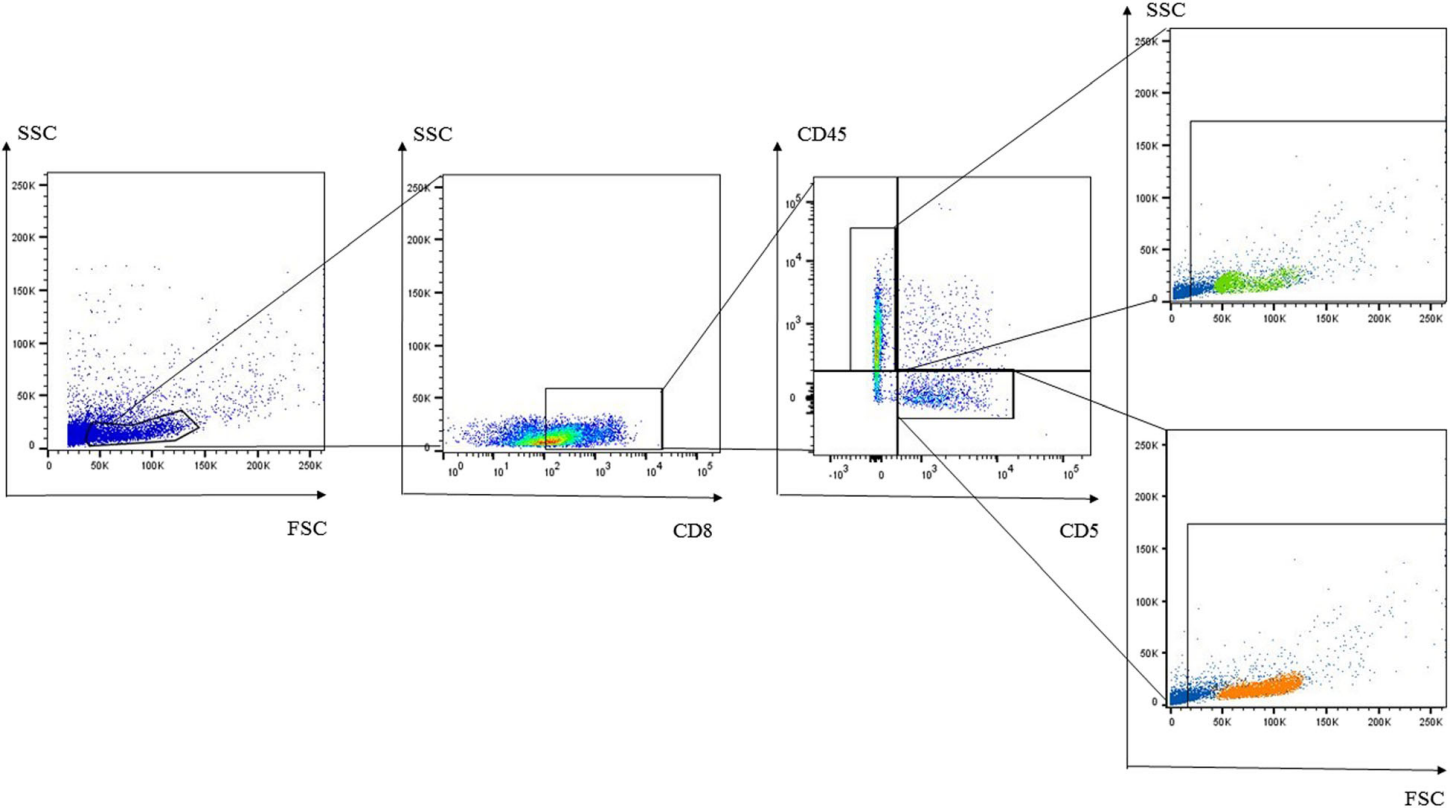
Representative flow cytometric dot plot of lion PBMCs. CD8 + CD5 + CD45- and CD8 + CD5-CD45+ lymphocytes were selected for back gating to establish their distribution across the two distinct lymphocyte populations observed on the FSC versus SSC dot plots

The two distinct PBMC lymphocyte populations differed both in size and antibody staining characteristics. The smaller lymphocytes were mainly cells staining CD4^+^, whereas the larger lymphocytes were predominantly CD8^+^ cells (Fig. 2; Table 2). Small CD4^+^ cells were primarily CD5^−^/CD45^+^, while large CD4^+^ cells were primarily CD5^+^/CD45^−^ (Fig. 3, Table 3), which is similar to the pattern observed in CD8^+^ cells (Fig. 4, Table 4). The CD4:CD8 ratio in total lymphocytes in PMBCs of Lion 1, Lion 2 and the tiger were 3.5, 7.1 and 1.8, respectively.

**Table 2:** CD4 and CD8 expression (%) on small and large lion and tiger lymphocytes in PBMC sample at Day 1

	Total lymphocytes		Small lymphocytes		Large lymphocytes	
	CD4^+^	CD8^+^	CD4^+^	CD8^+^	CD4^+^	CD8^+^
Lion 1	51.9	14.8	69.0	5.06	14.5	21.5
Lion 2	52.9	7.5	48.3	4.60	7.03	12.5
Tiger	26.4	14.5	39.2	13.9	1.59	18.0

**Table 3:** CD5 and CD45 expression on small and large CD4^+^ cells in *Panthera* PBMCs (%) at Day 1 as determined by flow cytometry

	Small lymphocytes				Large lymphocytes			
	CD5^+^		CD5^−^		CD5^+^		CD5^−^	
	CD45^−^	CD45^+^	CD45^−^	CD45^+^	CD45^−^	CD45^+^	CD45^−^	CD45^+^
Lion 1	14.5	4.55	19.5	61.4	56.6	4.3	4.9	32.9
Lion 2	10.0	6.7	19.8	63.3	29.3	8.1	8.4	54.2
Tiger	46.2	13.8	3.28	36.8	62.1	1.5	2.2	34.2

**Table 4:** CD5 and CD45 expression on small and large CD8^+^ cells in *Panthera* PBMCs (%) at Day 1 as determined by flow cytometry

	Small lymphocytes				Large lymphocytes			
	CD5^+^		CD5^−^		CD5^+^		CD5^−^	
	CD45^−^	CD45^+^	CD45^−^	CD45^+^	CD45^−^	CD45^+^	CD45^−^	CD45^+^
Lion 1	19.0	18.1	3.7	59.2	49.5	7.43	8.8	34.3
Lion 2	33.9	24.2	7.37	34.5	40.9	16.4	9.6	33.0
Tiger	62.9	19.9	7.7	9.52	78.4	7.89	4.2	9.6

The predominant CD4^+^ subsets identified in the small and large lymphocytes were CD5^−^CD45^+^ and CD5^+^CD45^−^ respectively. These populations were gated on in both PBMCs (small and larger lymphocytes combined) and RCLLs to analyse CD25^+^ and CD44^+^ expression. In the PBMC sample, 48.4% of the CD4^+^CD5^+^CD45^−^ cells were CD25^+,^ however this pattern was not mirrored in the RCLL sample (Additional file 1: Table S1). CD44^+^ cells were observed in both PBMCs and RCLLs, but the expression varied greatly.

**Fig. 5:**
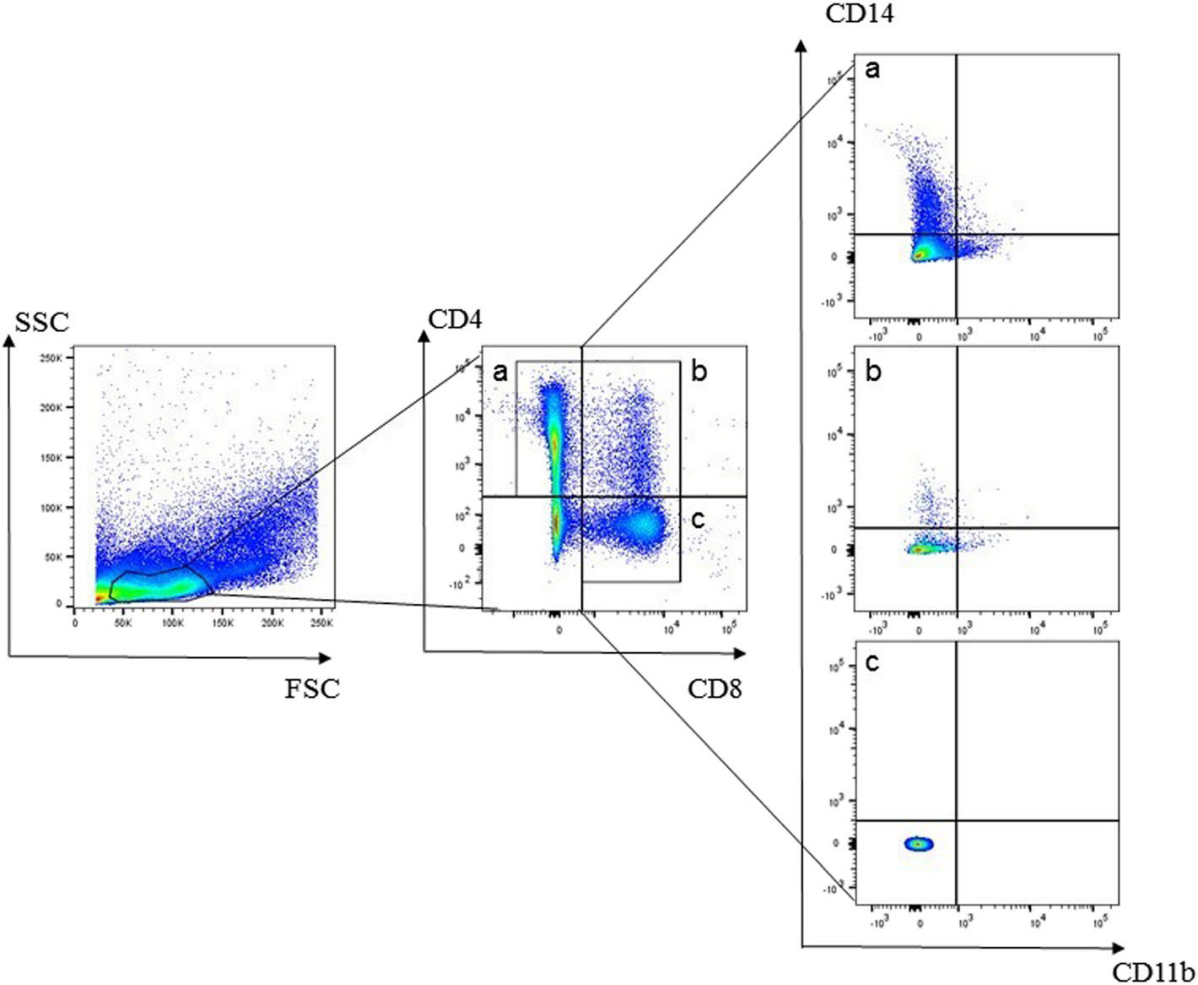
Flow cytometric dot plot of peripheral blood cell sorting. CD4 + CD8- (**a**), CD4 + CD8+ (**b**) and CD4-CD8+ (**c**) lymphocyte subsets were selected to evaluate CD14 and CD11b expression

Expression of CD14 and CD11b were restricted to lymphocytes that were CD4^+^, while CD8^+^ lymphocytes showed no evidence of CD14 or CD11b expression. Moreover, CD14 and CD11b were primarily expressed on different CD4^+^ sub-populations (Fig. 5, Table 5).

**Table 5:** CD14 and CD11b expression (%) on CD 4^+^ and CD8^+^ cells in *Panthera* PBMCs at Day 1 as determined by flow cytometry

	CD4^+^CD8^−^		CD4^+^CD8^+^		CD4^−^CD8^+^	
	CD14^+^	CD11b^+^	CD14^+^	CD11b^+^	CD14^+^	CD11b^+^
Lion 1	4.56	2.34	3.92	0	0	0
Lion 2	3.86	0.97	1.55	3.10	0	0
Tiger	5.63	2.33	0.56	0	0	0

### Influence of blood processing technique on staining pattern

CD4 and CD8 antibody staining was comparable in the PBMC and RCLL samples of both lions and the tiger. The staining of CD25, CD14 and CD11b all showed large differences between PBMC and RCLL samples for the two lions, but were comparable for the tiger (Additional file 1: Table S2).

### Effect of time delay on cell surface staining

The expression of surface markers over time varied greatly between animals, with relatively stable expression over Day 1 and Day 2 in some markers (i.e. Lion 1 PBMC CD4^+^ expression) but not in others (i.e. Lion 1 PBMC CD25^+^ expression) (Additional file 1: Tables S3, S4 and S5). The most stably observed populations were both PBMC and RCLL CD8^+^ cells, as well as CD4^+^CD5^−^CD45^+^ PBMCs.

## Discussion

Lymphocytes from both lions and a tiger, isolated by centrifugation over a density gradient, separated into distinct small and large populations. This phenomenon has previously been described in domestic cats [13], but not in lions [8–10, 14]. The smaller lymphocytes were predominantly characterized as CD4^+,^ whereas the larger lymphocyte population was mostly CD8^+^ cells. This differs from what is seen in domestic cat lymphocytes, which have a higher proportion of CD8^+^ cells in the small lymphocyte population [13]. In the total PBMC lymphocyte population the CD4:CD8 ratio varied greatly between the three animals, with both lions having similar or higher ratios than previously reported for healthy African lions [10, 15]. Unlike previous reports of distinct CD8β dim and bright cells observed in [9, 10], these subsets were not detected in the present study.

In the present study, CD45, classically known as a pan-lymphocyte marker [16, 17], did not react with all *Panthera* lymphocyte populations. Studies have reported that CD45R is found on B cells, T cells and thymocytes [18]. The expression of CD45R is particularly noted on activated murine T cells [18, 19]. As T lymphocytes express various forms of the leukocyte-common antigen CD45 [19], it is plausible that the CD45- lymphocytes express another isoform of CD45 that was not detected. Additionally, on CD4^+^ and CD8^+^ lymphocytes, CD5 and CD45 did not co-localize, with the smaller lymphocytes predominantly being CD5^−^CD45^+^ and the larger lymphocytes CD5^+^CD45^−^. Other authors have considered CD5^−^ lion lymphocytes as B cells [10], neglecting the possibility of species specific differences in immune cell surface receptor expression as observed in adult rabbits where all peripheral B lymphocytes express CD5 [20]. This may have led to an over or underestimation of the true percentage of CD4^+^ lymphocytes, as only CD5^+^ lymphocytes were considered, excluding any cells which may be CD5^−^CD4^+^. Previous studies have described cytotoxic CD3^+^CD5^−^CD4^+^ cells in humans [21] and activated CD5^−^CD4^+^ and CD5^−^CD8^+^ cells in ovine lentivirus infected sheep [22]. In *Panthera* species, markers of activation may be similar, and the expression of CD5, CD45 and CD4 should be investigated further.

Evidence for a highly stable subset of CD8^+^CD5^−^ cells has been described in humans [23]. These cells are a major source of lymphotactin which is a chemotactic agent for lymphocytes and may play a role in immune regulation [23]. In the present study the majority of CD8^+^ lymphocytes were identified as CD8^+^CD5^−^CD45^+^ or CD8^+^CD5^+^CD45^−^, which has not been previously reported in studies characterizing the immunophenotype of lion cells [8–10, 14]. These cells may play an important role in immune regulation and should be investigated further.

The co-expression of CD4 and CD14 on the surface of lion lymphocytes was unexpected. Although these may be CD4^+^CD14^+^ lymphocytes, CD4^+^CD14^+^ macrophages have been observed in both humans and rats [24, 25]. These macrophages have been described to play a role in HIV pathogenesis [25] and may be involved in immune responses to FIV in lions. CD8^+^CD14^+^ lymphocytes have been identified in humans, but not murine, lymphocytes after stimulation and these are able to secrete high levels of interferon gamma, granzyme and perforin [25]. In the present study there was no evidence of CD14 or CD11b expression on CD8^+^ cells, however both CD4^+^CD11b^+^ and CD4^+^CD14^+^ lymphocytes were observed. The CD11b adhesion protein is associated with T cell activation, and its expression on T cells has been associated with persistent bacterial infection [25]. Conversely, CD11b expression on CD8^+^ cells are characteristic of viral infections [26]. The current study is the first to describe the expression of CD14 and CD11b on lion lymphocytes. However, a study investigating the effects of FIV on T lymphocyte populations described the expression of CD18, which associates with the 11b integrin chain [27]. Higher expression of CD18, defined as a marker of activation, has also been observed on CD8^+^ T cells in FIV-positive lions [9], supporting the increased expression of integrins on virally activated cells. These markers should be investigated further, and may provide insights into the mechanism of immune regulation in *M. bovis* or FIV infection.

Previous immunophenotyping studies using lion peripheral blood have investigated the influence of FIV on CD44 and CD49 as activation markers [9]. In the present study however, we describe the novel identification of CD4^+^CD25^+^ cells in lion PBMCs and RCLLs, with staining patterns similar to that of regulatory T cells in domestic cats [28]. Lion CD44^+^ lymphocytes have also been characterized in the present study using a canine cross-reactive clone, IM7. A previous report described the use of CD44 clone MEM85 in lions, and found CD44 expression to remain unchanged by FIV [9]. The genetic sequence coding for these two clones have been found to be homoglous with different regions of the CD44 gene [29]. Furthermore, the antibody from the MEM85 clone is reported to bind to human CD44, but not canine CD44, whereas the antibody from the IM7 clone was described to be cross-reactive with mouse, dog, non-human primates and humans [29]. CD44 is essential for the generation of memory Th1 cells [30] and also plays a role in cell adhesion and migration, lymphocyte homing, activation and proliferation [31]. In FIV-infected domestic cats, Th1 immune responses have been shown to inhibit virus replication [32]. Investigating the co-localization of IM7 and MEM85 may increase confidence and improve insight into the effects of FIV on the CD44^+^ directed immune activation in lion lymphocytes.

Due to the remote locations where wild lion populations are found as well as the harsh in-field conditions, the influence of both time to processing as well as the influence of different sampling methods were investigated. The effects of sample processing varied greatly between the animals, with CD4 and CD8 antibody staining being comparable between the PBMC and RCLL samples of both lions and the tiger. Although staining patterns using CD25, CD14 and CD11b in PBMCs and RCLLs were similar in the tiger, they were not in the lions. Previous reports of flow cytometric analysis of T-lymphocyte subsets in cats report that fluorescence intensity was not adversely affected by the whole blood lysis technique [12]. Moreover, the influence of sample processing time on antibody binding was inconclusive, with highly variable patters observed. Further investigation in a larger cohort of animals is required to establish the usefulness of the whole blood lysis technique in lion whole blood, as well as the influence of processing time on antibody binding to cell surface markers. Further validation of the reactivity of these antibodies in *Pathera* species using transcriptomics will prove useful in confirming and clarifying the lymphocyte subsets observed. Moreover, as FIV infection has been reported to influence the distribution of small and large lymphocytes in domestic cats [13], it may be useful to further investigate lymphocyte activation in these populations in infected lions.

The role of T-lymphocytes in the lion immune response are poorly understood. To our knowledge, this is the first published report on the flow cytometric analysis of tiger lymphocytes and the use of these commercially obtained antibodies in lion RCLLs. Additionally, we describe the presence of possible activated subsets within these samples which may give insight into the mechanism of immune protection during pathogenic infections. A number of previous studies have investigated lion lymphocyte populations by using commercially available antibodies developed for the domestic cat [8–10, 14, 33], demonstrating the conserved nature of cell surface epitopes among felids and the usefulness of using domestic cat-specific antibodies in lions [8].

## Conclusion

This study shows that commercially obtained antibodies are able to recognise cell surface markers in both lion PBMCs and RCLLs, and that there is substantial loss of signal over time, advocating for the timely processing of samples for immunophenotyping. This paper provides a platform which may enable future immune-phenotyping studies to investigate the immune response to FIV and *M. bovis* infections, pathogens commonly affecting the lion populations in South Africa.

## Methods

### Animals and sample collection

**Fig. 6:**
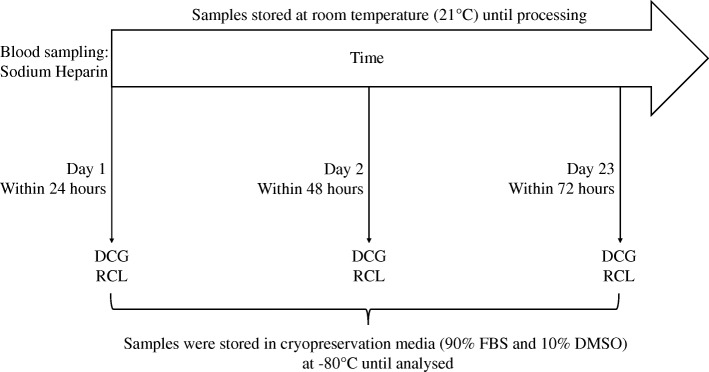
Whole blood was collected in sodium heparin tubes and stored at room temperature until processed. Samples were processed on Day 1 (within 24 h of collection), Day 2 (within 48 h of collection) and Day 3 (within 72 h of collection), by both red cell lysis (RCL) of the whole blood and density gradient centrifugation (DGC). The processed samples were stored in cryomedia, containing foetal bovine serum (FBS) and dimethyl sulfoxide (DMSO), at − 80 °C

Two captive lions (*Panthera leo)* and one captive tiger (*Panthera tigris*) were opportunistically sampled from a private game reserve in the Free State, South Africa, during routine health examinations. Blood samples were collected by jugular venipuncture into sodium heparin blood collection tubes and stored at room temperature for 1, 2 and 3 days, respectively (Fig. 6). Approval for this study was obtained from the Stellenbosch University Animal Care and Use Committee (SU-ACUD15–00013). Permission to use the blood obtained from these animals was granted bu. the Head of Animal Welfare at LionsRock Big Cat Sanctuary.

### Blood processing

Two sample processing methods to obtain lymphocytes from whole blood were compared in order to establish whether antibody binding or cell surface receptor expression and detection were influenced by either method.

### Red cell lysis of whole blood

Five hundred microliter aliquots of whole blood harvested on Day 1, 2 and 3 were incubated with 1 ml of Red Cell Lysis Buffer (BD Bioscience, San Jose, CA, USA) in the dark for 10 min, at room temperature. Cells were harvested by centrifugation at 450 x g for 5 min and washed three times with flow cytometry staining buffer (phosphate-buffered saline (PBS) containing 2% foetal bovine serum (FBS) (Capricorn Scientific, Ebsdorfergrund, Germany), then resuspended and stored in cryopreservation media consisting of 90% FBS and 10% dimethyl sulfoxide (DMSO, Sigma-Aldrich, St. Louis, MO, USA) as three aliquots at − 80 °C. Cell staining was performed within 6 months. Samples obtained using this method will hereafter be referred to as red cell lysis leukocytes (RCLLs).

### Density gradient centrifugation

On Day 1, 2 and 3, 10 ml of whole blood was diluted 1:1 with sterile PBS and layered on 20 ml of Ficoll-Paque (GE Healthcare Bio-Sciences, Uppsala, Sweden). Samples were centrifuged for 25 min at 400×g, without applying a brake. The peripheral blood mononuclear cell (PBMC) interface was carefully aspirated and cells were washed twice with PBS by centrifugation for 10 min at 400×g. The PBMCs were resuspended in residual PBS and stored in cryopreservation media as three aliquots at − 80 °C until staining was performed. Cell staining was performed within 6 months. Samples obtained using this method will hereafter be referred to as PBMCs.

### Cell surface receptor staining

Cells stored at − 80 °C were thawed in a water bath at 37 °C and washed twice with staining buffer before being resuspended in 4 ml staining buffer. During initial optimization, 50 μl of resuspended cells were incubated with dilutions of fluorophore-labelled antibody (1:5, 1:10, 1:25, 1:50, 1:100 and 1:200), with final concentrations of antibody at 0.25–5 μg/ml (Table 1). The optimal antibody concentration was determined as previously described [9]. The cells were stained at the optimal concentration for 60 min at 4 °C in the dark, with manual agitation 30 min into the staining protocol. After staining, cells were washed according to manufacturer’s instructions. A FACS Canto II (BD Bioscience) was used for cell acquisition (≥10,000 events). The instrument was calibrated and setup according to the manufacturer’s instructions. Quality controls included the use of Rainbow Beads (eBioscience—San Diego, CA, USA) and BD™ CompBeads to adjust the compensation settings accordingly.

### Gating strategy

In order to identify lymphocyte populations, doublets were excluded using forward scatter (FSC) area versus FSC height as previously described [11]. Following exclusion of debris, lymphocyte subsets were identified and other cell types, including monocytes, excluded based on FSC and side scatter (SSC) characteristics, as for other species [11, 12]. From this gate, CD4^+^, CD4^−^, CD8^+^ or CD8^−^ T lymphocytes were selected for further analysis depending on the panel analysed. The PBMCs and RCLLs were stained with three different antibody combinations: Panel 1: CD5, CD4, CD25, CD44, CD45; Panel 2: CD4, CD8, CD14, CD11b; and Panel 3: CD 5, CD8, CD45. The initial strategy first gated on all CD45+ cells (as is the standard approach), however due to large proportion of the lymphocytes being excluded from the analysis, as well as the observation of a large subset of CD4+ cells which did not express CD45, the gating strategy was changed to the above. Based on our current understanding of the human immune system we do not expect CD4+ cells to not express CD45, but due to species specific differences observed by other authors [20] this possibility in lions cannot be ruled out.

### Data and statistical analysis

All data were analysed using FlowJo Version 10 software (Tree Star, Ashland, OR, USA). Nonparametric tests (Mann-Whitney U tests) were used to compare antibody staining patterns over time. A *p-*value of 0.05 or less was considered significant. All statistical analyses were performed using GraphPad Prism version 5.00 (GraphPad Software, Inc., La Jolla, CA, USA).

## Additional file

Additional file 1: **Figure S1.** Staining profiles for commercially available antibodies a) CD4, b) CD8, c) CD44, d) CD5, e) CD45, f) CD25, g) CD14 and h) CD11b that showed evidence of cross-reactivity with *Panthera* lymphocytes in the PBMC sample. **Figure S2.** Staining profiles for commercially available antibodies a) CD4, b) CD8, c) CD44, d) CD5, e) CD45, f) CD25, g) CD14 and h) CD11b that showed evidence of cross-reactivity with *Panthera* lymphocytes in the RCLL sample. **Table S1.** CD44 and CD25 expression on CD4 + CD5 + CD45- and CD4 + CD5-CD45+ lymphocytes in PBMCs and RCLLs (%) at Day 1. **Table S2.** Expression of cell surface markers (%) in *Panthera* species PBMCs versus RCLLs on Day 1. **Table S3.** Cell surface marker expression (%) on lymphocytes in PBMC and RCLL samples of Lion 1 over time. **Table S4.** Cell surface marker expression (%) on lymphocytes in PBMC and RCLL samples of Lion 2 over time. **Table S5.** Cell surface marker expression (%) on lymphocytes in PBMC and RCLL samples of the tiger over time. (PDF 456 kb)

## Abbreviations

DMSO: Dimethyl sulfoxide
FBS: Foetal bovine serum
FIV: Feline immunodeficiency virus
FSC: Forward scatter
PBMC: Peripheral blood mononuclear cell
PBS: Phosphate buffered saline
RCLL: Red cell lysis lecukocyte
SSC: Side scatter
