# Supplementary material for: A pilot study evaluating the utility of commercially available antibodies for flow cytometric analysis of Panthera species lymphocytes
Source: BMC Vet Res. 2018 Dec 19;14:410. doi: 10.1186/s12917-018-1717-4 (PMC6299994; doi:10.1186/s12917-018-1717-4)
Supplement: Supplementary file 1 — Figure S1. Staining profiles for commercially available antibodies a) CD4, b) CD8, c) CD44, d) CD5, e) CD45, f) CD25, g) CD14 and h) CD11b that showed evidence of cross-reactivity with Panthera lymphocytes in the PBMC sample. Figure S2. Staining profiles for commercially available antibodies a) CD4, b) CD8, c) CD44, d) CD5, e) CD45, f) CD25, g) CD14 and h) CD11b that showed evidence of cross-reactivity with Panthera lymphocytes in the RCLL sample. Table S1. CD44 and CD25 expression on CD4 + CD5 + CD45- and CD4 + CD5-CD45+ lymphocytes in PBMCs and RCLLs (%) at Day 1. Table S2. Expression of cell surface markers (%) in Panthera species PBMCs versus RCLLs on Day 1. Table S3. Cell surface marker expression (%) on lymphocytes in PBMC and RCLL samples of Lion 1 over time. Table S4. Cell surface marker expression (%) on lymphocytes in PBMC and RCLL samples of Lion 2 over time. Table S5. Cell surface marker expression (%) on lymphocytes in PBMC and RCLL samples of the tiger over time. (PDF 456 kb) [file 12917_2018_1717_MOESM1_ESM.pdf]

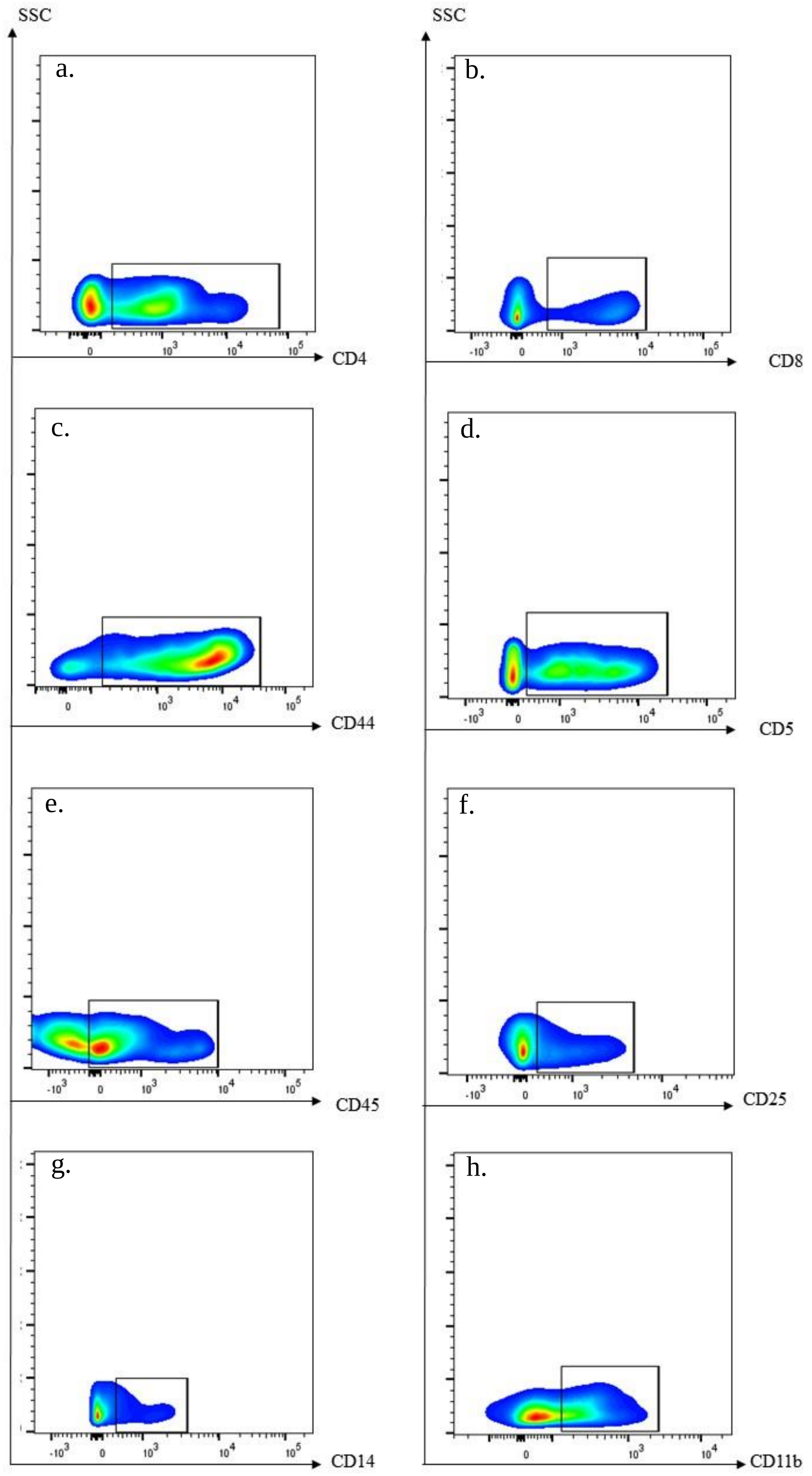

Figure S1 Staining profiles for commercially available antibodies a) CD4, b) CD8, c) CD44, d) CD5, e) CD45, f) CD25, g) CD14 and h) CD11b that showed evidence of cross-reactivity with *Panthera* lymphocytes in the PBMC sample.

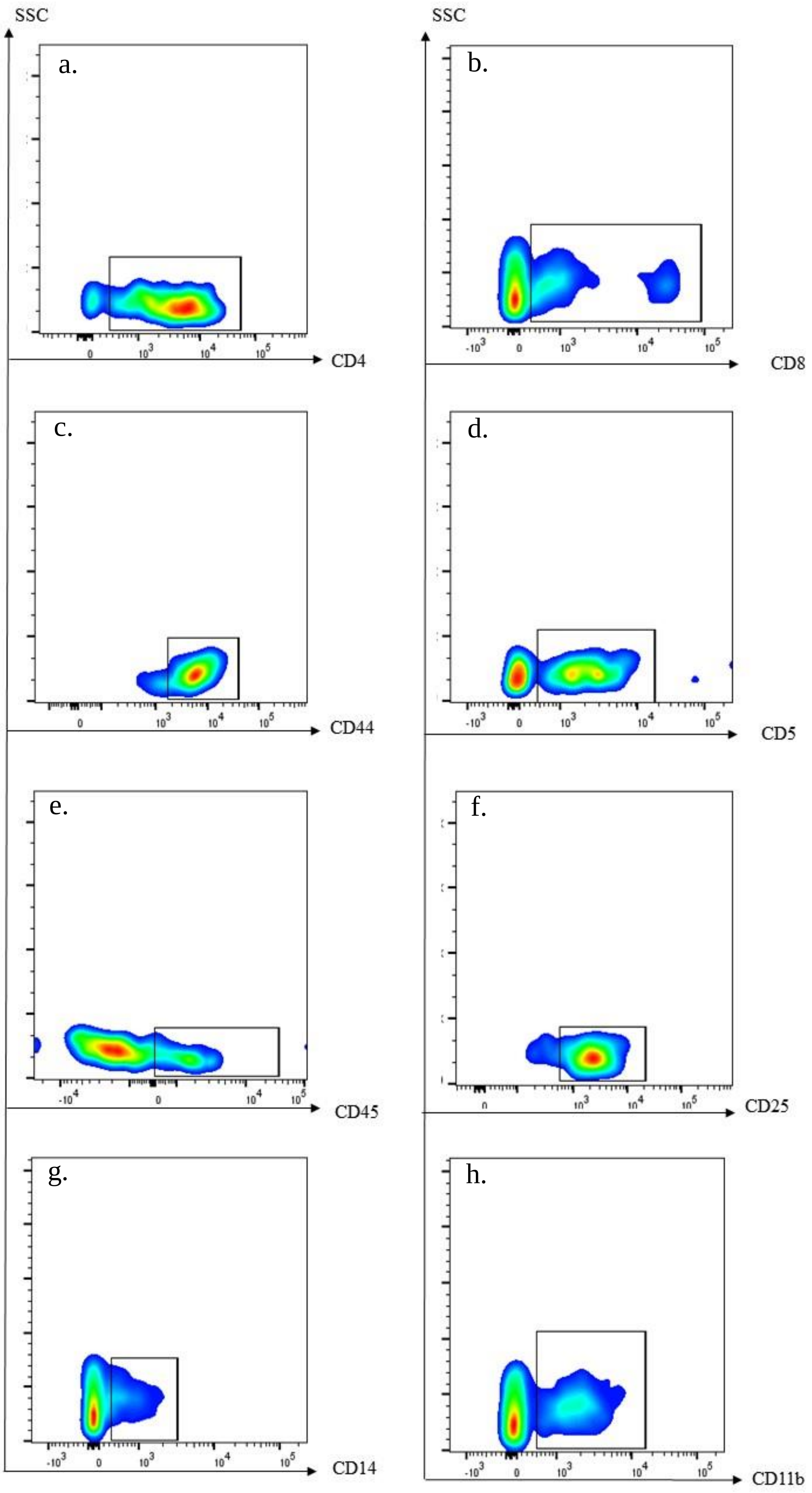

Figure S2 Staining profiles for commercially available antibodies a) CD4, b) CD8, c) CD44, d) CD5, e) CD45, f) CD25, g) CD14 and h) CD11b that showed evidence of cross-reactivity with *Panthera* lymphocytes in the RCLL sample.

Table S1 CD44 and CD25 expression on CD4+CD5+CD45- and CD4+CD5-CD45+ lymphocytes in PBMCs and RCLLs (%) at Day 1.

		Lion 1		Lion 2		Tiger	
		PBMC	RCLL	PBMC	RCLL	PBMC	RCLL
CD4+CD5+ CD45-	CD44+CD25-	14.9	20.6	8.84	11.1	5.15	21.1
	CD44-CD25+	48.4	0	38.2	0	37.9	0
	CD44+CD25+	5.38	79.4	5.35	88.9	2.03	78.9
CD4+CD5-CD45+	CD44+CD25-	1.95	14.3	4.13	3.33	8.92	12.5
	CD44-CD25+	9.02	7.79	6.02	0	20.8	3.12
	CD44+CD25+	0.57	76.6	0.38	96.7	4.18	84.4

Table S2 Expression of cell surface markers (%) in *Panther* *a* species PBMCs versus RCLLs on Day 1.

	Lion 1		Lion 2		Tiger	
	PBMC	RCLL	PBMC	RCLL	PBMC	RCLL
CD8+	16.3	20.2	10.3	11.3	18.4	18.5
CD4+	50.6*	60.3*	59.0	63.9	34.6	32.5
CD4+CD25+	16.8	5.80	5.36	12.1	21.0	29.3
CD4+CD14+	7.34	11.5	6.95	30.7	10.8	11.8
CD4+CD11b+	1.86	0.00	0.62	0.00	1.50	2.35

*****Values indicated for Lion 1 CD4+ are for Day 2.

Table S3 Cell surface marker expression (%) on lymphocytes in PBMC and RCLL samples of Lion 1 over time.

			CD4+		CD4+CD5+CD45-			CD4+CD5-CD45+			CD4+	
PBMC	CD4+	CD8+	CD5+CD45-	CD5-CD45+	CD44+	CD25+	CD44+CD25+	CD44+	CD25+	CD44+CD25+	CD11b	CD14
Day 1	50.8	16.3	16.2	70.3	14.9	48.4	5.38	1.95	9.02	0.57	1.86	7.49
Day 2	50.6	12.0	13.3	76.0	22.7	28.1	6.28	1.70	3.02	0.27	0.94	5.61
Day 3	44.1	15.5	18.8	60.1	15.2	38.2	5.83	3.30	10.9	1.04	3.01	6.37
RCLL	CD4+	CD8+	CD5+CD45-	CD5-CD45+	CD44+	CD25+	CD44+CD25+	CD44+	CD25+	CD44+CD25+	CD11b	CD14
Day 1	13.3	20.2	19.6	22.2	20.6	0	79.4	14.3	7.79	76.6	0	11.5
Day 2	60.3	18.1	28.6	31.6	22.7	0	77.3	12.5	4.69	81.2	0	26.7
Day 3	46.5	19.2	16.1	28.9	5.88	0	94.1	6.56	4.92	86.0	0	23.3

Table S4 Cell surface marker expression (%) on lymphocytes in PBMC and RCLL samples of Lion 2 over time.

			CD4+		CD4+CD5+CD45-			CD4+CD5-CD45+			CD4+	
PBMC	CD4+	CD8+	CD5+CD45-	CD5-CD45+	CD44+	CD25+	CD44+CD25+	CD44+	CD25+	CD44+CD25+	CD11b	CD14
Day 1	59.0	10.3	11.9	61.4	8.84	38.2	5.35	4.13	6.02	0.38	0.62	6.95
Day 2	47.8	12.1	8.19	60.4	15.9	22.6	4.88	2.54	5.63	0.56	0.95	6.64
Day 3	40.0	10.5	13.5	58.4	8.05	45.5	5.23	3.45	13.4	0.56	0.27	14.2
RCLL	CD4+	CD8+	CD5+CD45-	CD5-CD45+	CD44+	CD25+	CD44+CD25+	CD44+	CD25+	CD44+CD25+	CD11b	CD14
Day 1	59.6	11.3	21.8	48.0	11.1	0	88.9	3.33	0	96.7	0	30.7
Day 2	41.2	13.2	13.0	52.1	0	0	100	1.74	8.70	89.6	0	50.0
Day 3	37.3	8.55	19.6	56.1	3.12	0	96.7	3.61	7.23	89.2	0	29.3

Table S5 Cell surface marker expression (%) on lymphocytes in PBMC and RCLL samples of the tiger over time.

			CD4+		CD4+CD5+CD45-			CD4+CD5-CD45+			CD4+	
PBMC	CD4+	CD8+	CD5+CD45-	CD5-CD45+	CD44+	CD25+	CD44+CD25+	CD44+	CD25+	CD44+CD25+	CD11b	CD14
Day 1	34.6	18.4	37.7	28.0	5.15	37.9	2.03	8.92	20.8	4.18	1.50	10.9
RCLL	CD4+	CD8+	CD5+CD45-	CD5-CD45+	CD44+	CD25+	CD44+CD25+	CD44+	CD25+	CD44+CD25+	CD11b	CD14
Day 1	32.5	18.5	25.0	40.0	21.1	0	78.9	12.5	3.12	84.4	2.35	11.8
Day 2	14.4	8.76	30.4	22.0	0	0	100	0	0	100	0	34.6
Day 3	20.2	3.53	14.5	43.4	100	0	0	91.7	0	0	0	42.2
